# Anti-Inflammatory and Vasorelaxant Effects Induced by an Aqueous Aged Black Garlic Extract Supplemented with Vitamins D, C, and B12 on Cardiovascular System

**DOI:** 10.3390/foods12071558

**Published:** 2023-04-06

**Authors:** Lucia Recinella, Maria Loreta Libero, Valentina Citi, Annalisa Chiavaroli, Alma Martelli, Roberta Foligni, Cinzia Mannozzi, Alessandra Acquaviva, Simonetta Di Simone, Vincenzo Calderone, Giustino Orlando, Claudio Ferrante, Serena Veschi, Anna Piro, Luigi Menghini, Luigi Brunetti, Sheila Leone

**Affiliations:** 1 Department of Pharmacy, G. d’Annunzio University of Chieti-Pescara, 66013 Chieti, Italy; 2 Department of Pharmacy, University of Pisa, 56126 Pisa, Italy; 3 Interdepartmental Research Center “Nutrafood: Nutraceutica e Alimentazione per la Salute”, University of Pisa, 56126 Pisa, Italy; 4 CISUP, Centre for Instrumentation Sharing of Pisa University, 56126 Pisa, Italy; 5 Department of Agricultural, Food and Environmental Sciences, Polytechnic University of Marche, Via Brecce Bianche 10, 60121 Ancona, Italy; 6 Veridia Italia Srl, Via Piano di Sacco, 65013 Città Sant’Angelo, Italy

**Keywords:** aged black garlic, hydrogen sulfide, oxidative stress, inflammation

## Abstract

Multiple studies demonstrated biological activities of aged black garlic, including anti-inflammatory, antioxidant, and cardioprotective effects. We aimed to investigate the protective effects of an aged black garlic water extract (ABGE) alone or in association with multivitamins consisting of combined Vitamins D, C, and B12, on mouse heart specimens exposed to E. coli lipopolysaccharide (LPS). Moreover, we studied the hydrogen sulphide (H_2_S) releasing properties and the membrane hyperpolarization effect of the Formulation composed by ABGE and multivitamins, using Human Aortic Smooth Muscle Cells (HASMCs). ABGE, vitamins D and C, and the Formulation suppressed LPS-induced gene expression of cyclooxygenase (COX)-2, tumor necrosis factor (TNF)-α, interleukin (IL)-6, nuclear factor-kB (NF-kB), and inducible nitric oxide synthase (iNOS) on mouse heart specimens. The beneficial effects induced by the extract could be related to the pattern of polyphenolic composition, with particular regard to gallic acid and catechin. The Formulation also increased fluorescence values compared to the vehicle, and it caused a significant membrane hyperpolarization of HASMCs compared to ABGE. To conclude, our present findings showed that ABGE, alone and in association with multivitamins, exhibited protective effects on mouse heart. Moreover, the Formulation increased intracellular H_2_S formation, further suggesting its potential use on cardiovascular disease.

## 1. Introduction

Cardiovascular disease (CVD) represents the main cause of mortality all over the world. In this context, it has been demonstrated that inflammation and oxidative stress play a pivotal role in the development of CVD such as heart failure, acute coronary syndromes, atherosclerosis, and hypertension [1,2,3,4]. In agreement, an association between alterations in levels of pro-inflammatory and pro-oxidant markers and CVD has been found [5]. Various herbal extracts, particularly in pharmacological associations, were shown to be effective in decreasing the burden of inflammation and oxidative stress [6,7,8,9]. Moreover, hydrogen sulfide (H_2_S), a gaseous molecule, is critically involved in CVD. To this end, several preclinical and clinical studies suggested its protective role in heart failure, myocardial infarction, and hypertension [10].

Aged black garlic (ABG), obtained from fresh garlic (*Allium sativum* L.) and fermented under high temperatures (60–90 °C) and high humidity (80–90%) for a specific time period, exerts beneficial effects in various experimental paradigms. In particular, ABG induced multiple biological activities, including antioxidant, antiallergic, antidiabetic, anti-inflammatory, cardiovascular, hepatoprotective, neuroprotective, and anticarcinogenic effects [11,12,13,14,15,16,17,18,19]. The protective effects induced by aged garlic on different types of CVD have been demonstrated in several studies [20]. In agreement, aged garlic was found able to decrease high blood pressure in humans [21]. Furthermore, black garlic extract was found able to modify serum levels of triglycerides and cholesterol [12]. Various phytoconstituents, including phenolics, S-allyl cysteine (SAC), and hydroxycinnamic acid derivatives were found in BG, with respect to raw garlic [12,13].

The beneficial properties of garlic in CVD have been hypothesized to be related to polyphenolic compounds and SAC [22]. Multiple studies showed significant differences in the total phenolic content of black and fresh garlic. In particular, the content in phenolic compounds is 5–8-times higher in black garlic than that in fresh garlic [23].

The maintenance of cardiovascular health is also attributed to vitamins, such as vitamin B 12, vitamin C, and vitamin D [24].

In particular, vitamin D supplementation improves the cardiac function [25], while vitamin C supplementation, thanks to its antioxidant properties, is effective in the prevention or treatment of several cardiovascular diseases [26]. Furthermore, vitamin B12 deficiency promotes the onset of different CVD, including myocardial infarction, stroke, and other circulatory health problems [27].

The present study aims to investigate the potential antioxidant and anti-inflammatory effects of an ABG water extract (ABGE), alone or in association with multivitamins consisting of the combined Vitamin D, C, and B12 Formulation on mouse heart specimens exposed to *Escherichia coli* lipopolysaccharide (LPS), a known proinflammatory agent. In this context, we evaluated gene expression of various biomarkers involved in inflammation and oxidative stress, including cyclooxygenase (COX)-2, tumor necrosis factor (TNF)-α, interleukin (IL)-6, nuclear factor-kB (NF-kB), and inducible nitric oxide synthase (iNOS). In addition, we studied the H_2_S releasing properties and the membrane hyperpolarization effect of ABGE, as well as the Formulation composed by ABGE and multivitamins using Human Aortic Smooth Muscle Cells (HASMCs).

The ABGE was also investigated in order to identify and quantify the polyphenolic content using high-performance liquid chromatography coupled with a photo diode array detector (HPLC-DAD) analytical method.

## 2. Materials and Methods

### 2.1. Preparation of ABGE

ABG cloves were supplied as dried material by il Grappolo S.r.l. (Soliera, Modena, Italy). Preparation of ABGE was performed as previously reported [28,29]. The detailed protocol is enclosed as supplementary materials.

### 2.2. Total Polyphenol Content of ABGE

Total polyphenol content was determined according to the Folin-Ciocalteu method, as described in Savini et al. (2017) [30] with some modifications. The detailed protocol related to total polyphenol content of ABGE is described in the Supplementary Materials Section.

### 2.3. HPLC-DAD-MS Analysis of Phenolic Compounds

The extract was analyzed for phenol quantitative determination using a reversed-phase HPLC-DAD-MS in gradient elution mode [31]. The details of the analysis are reported in Supplementary Materials (Tables S1 and S2).

### 2.4. Toxicological and Pharmacological Studies

#### 2.4.1. Cell Line

H9c2 cells (rat cardiomyoblasts, ATTC, Rockville, MD, USA) were maintained in DMEM (Sigma-Aldrich, St. Louis, MO, USA), supplemented with 10% fetal bovine serum (FBS), 1% of 100 unit/mL penicillin, and 100 mg/mL streptomycin (Sigma-Aldrich, St. Louis, MO, USA) in T75 red cap tissue culture flasks, at 37 °C in a humidified atmosphere of 5% CO_2_.

#### 2.4.2. Cell Viability Assay

Cell viability was evaluated by MTT assay [3-(4,5-dimethyl-2-thiazolyl)-2,5-diphenyl-2H-tetrazolium bromide] (Sigma, St. Louis, MO, USA), as previously described [32]. Briefly, H9c2 cell line was seeded in 96-well plates (5 × 10^3^ cells/well) and it was pretreated with 10 µg/mL LPS for 24 h. Subsequently, both LPS-pretreated and not LPS-pretreated H9c2 cells were exposed to ABGE at various concentrations (1–100 µg/mL), or with vehicle (control) for a further 48 h. On the basis of results, we then performed a second set of experiments to evaluate the effects induced by the Formulation [ABGE (100 µg/mL) + Vitamin B12 (1 µg/mL) + Vitamin C (10 µg/mL) + Vitamin D (1 µg/mL)] and the vitamins alone [Vitamin B12 (1 µg/mL), Vitamin C (10 µg/mL) and vitamin D (1 µg/mL)] on H9c2 cell viability in both LPS- and not LPS-pretreatment. The detailed protocol is described in the Supplementary Materials section.

#### 2.4.3. Ex Vivo Studies

Adult C57/BL6 male mice (3-month-old, weight 20–25 g) were housed in Plexiglas cages (2–4 animals per cage; 55 × 33 × 19 cm) and maintained under standard laboratory conditions (21 ± 2 °C; 55 ± 5% humidity) on a 14/10 h light/dark cycle, with ad libitum access to water and normal laboratory chow (RMH-B diet, Arie Blok animal feed, Woerden, the Netherlands). Housing conditions and experimentation procedures were strictly in agreement with the European Community ethical regulations (EU Directive no. 63/2010) on the care of animals for scientific research. According to the recognized principles of “Replacement, Refinement and Reduction in Animals in Research”, heart specimens were obtained as residual material from vehicle-treated animals randomized in our previous experiments, approved by the local ethical committee (‘G. d’Annunzio’ University, Chieti, Italy) and Italian Health Ministry (Project no. 885/2018-PR).

After collection, isolated heart specimens were maintained in a humidified incubator with 5% CO_2_ at 37 °C for 4 h (incubation period) in a RPMI buffer with added bacterial LPS (10 µg/mL), as previously described [33,34]. During the incubation period, the tissues were treated with ABGE (1 µg/mL, 10 µg/mL, 100 µg/mL), the Formulation [ABGE (10 µg/mL) + Vitamin B12 (1 µg/mL) + Vitamin C (10 µg/mL) + Vitamin D (1 µg/mL)], and the vitamins alone [Vitamin B12 (1 µg/mL), Vitamin C (10 µg/mL) and vitamin D (1 µg/mL)].

Extraction of total RNA was performed from the heart specimens using TRI Reagent (Sigma-Aldrich, St. Louis, MO, USA), in agreement with the manufacturer’s protocol. Contaminating DNA was removed using 2 units of RNase-free DNase 1 (DNA-free kit, Ambion, Austin, TX, USA). Determination of gene expression of COX-2, IL-6, NF-kB, TNF-α, and iNOS was performed by quantitative real-time PCR using TaqMan probe-based chemistry, as previously reported [7,35]. The detailed protocol is described in the Supplementary Materials Section.

#### 2.4.4. Cell Line

HASMCs were cultured in Medium 231 (Life Technologies, Carlsbad, CA, USA) supplemented with a Smooth Muscle Growth Supplement (SMGS, Life Technologies, Carlsbad, CA, USA) and 1% of 100 units/mL penicillin and 100 mg/mL streptomycin (Sigma Aldrich, St. Louis, MO, USA) in tissue culture flasks at 37 °C in a humidified atmosphere and 5% CO_2_, as previously described [36,37]. Cells were split 1:2 twice a week and used until passage 18.

#### 2.4.5. Evaluation of H_2_S Release on HASMCs

After 24 h, to allow cell attachment, the medium was replaced and cells were incubated for 30 min in the buffer standard (HEPES 20 mM, NaCl 120 mM, KCl 2 mM, CaCl_2_·2H_2_O 2 mM, MgCl_2_·6H_2_O 1 mM, Glucose 5 mM, pH 7.4, at room temperature), as previously described [36,37]. The detailed experimental procedure is reported in the Supplementary Materials Section.

#### 2.4.6. Evaluation of the Membrane Hyperpolarizing Effects on HASMCs

After 24 h to allow cell attachment, the medium was replaced and cells were incubated for 1 h in the buffer standard containing the bisoxonol dye bis-(1,3-dibutylbarbituric acid) DiBac4(3) (Sigma Aldrich, St. Louis, MO, USA) 2.5 μM [38]. NS1619 (Sigma-Aldrich, St. Louis, MO, USA) 10 μM, a BK_Ca_ channel opener, was used as a reference drug. The ABGE (1–100 µg/mL), or the Formulation and the vitamins alone (Vitamin B12 1 µg/mL, Vitamin C 10 µg/mL and Vitamin D 1 µg/mL), were added to the cells, and the trends of fluorescence were followed for 35 min. The relative fluorescence decrease, linked to hyperpolarizing effects, was recorded every 2.5 min and was calculated as previously reported [38]. Six different experiments (*n* = 6) were performed.

### 2.5. Statistical Analysis

The data were analyzed by the licensed software GraphPad Prism version 6.0 (Graphpad Software Inc., San Diego, CA, USA). Analysis of means ± SEM for each experimental group was performed by one-way analysis of variance (ANOVA), followed by either the Newman-Keuls multiple comparison post hoc test or by the Bonferroni post hoc test [39]. The level of significance was set to 0.05. The Tukey-Kramer’s Honest Significant Difference (HSD) test was used to compare the mean polyphenol contents of the extracts.

## 3. Results and Discussion

### 3.1. Total Polyphenol Content of ABGE

The ABGE provided a yield equal to 21.91 mg GAE/g DM in phenolic components [extraction yields of polyphenolic compounds obtained in ABGE (mg GAE/g DM): means ± SEM, 21.91 ± 1.07]. In our experiments, the ABGE showed a yield comparable to those reported by Najman et al. [35] (2021). Water extracts from conventional and organic black garlic have shown a content in polyphenolic components between 13.64 and 17.24 mg GAE/g DM [40]. In particular, a higher content in polyphenols was shown in black compared to fresh garlic, which was suggested to be dependent on various factors, including the garlic aging process (time, temperature, and relative humidity) [40].

### 3.2. HPLC-DAD-MS Analysis

**Figure 1:**
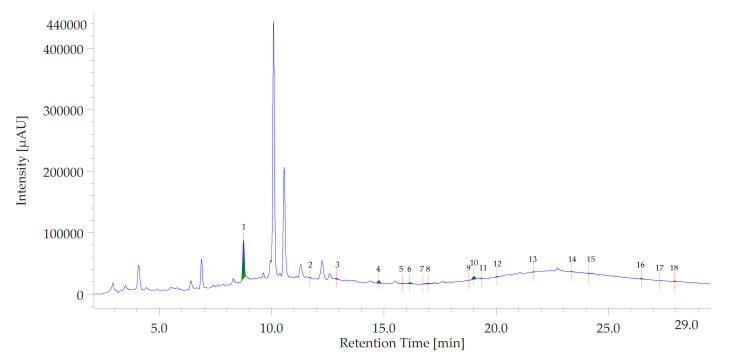
HPLC-DAD chromatogram of aged black garlic water extract (ABGE). The chromatographic analysis showed the presence of 12 phytochemicals: gallic acid (peak #1), 3-hydroxytyrosol (peak #2), caftaric acid (peak #3), catechin (peak #4), gentisic acid (peak #5), loganic acid (peak #7), chlorogenic acid (peak #8), caffeic acid (peak #10), epicatechin (peak #11), syringaldehyde (peak #13), benzoic acid (peak #16), and resveratrol (peak #18).

The retention times, m/z ratio, as well as quantity (μg/mL) of the investigated phenolic compounds in ABGE are reported in Table 1. In this context, a total of 12 compounds were identified at a wavelength of 254 nm. Gallic acid (#1) and catechin (#4) were the prominent phytochemicals, as shown in Figure 1.

Results are only in part comparable to those reported in the literature [41]. A study performed by Moreno-Ortega and collaborators [42] (2020) has found an increase in phenolic compounds, such as gallic acid and epigallocatechin gallate, in black compared to fresh garlic. In addition, it is well known that each cultivar expresses a different analytes content dependent on cultivation methods. Different studies have confirmed that the bioactive compounds of ABG possess a wide range of pharmacological activities, such as hypolipidemic, anticancer, and cardiovascular effects [43], which have been suggested to be mainly due to its anti-inflammatory and antioxidant properties.

### 3.3. Toxicological and Pharmacological Studies

**Figure 2:**
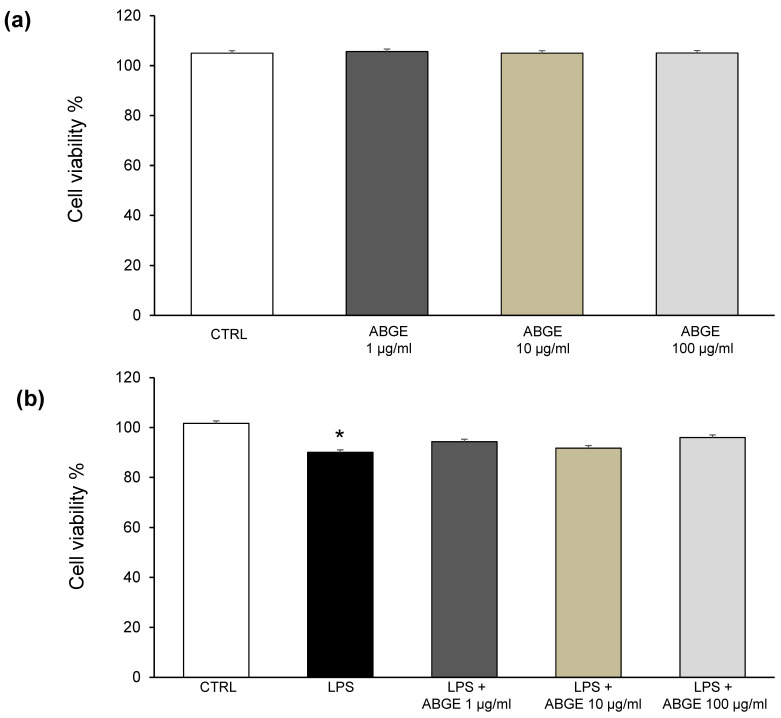
MTT assay of H9c2 cells exposed to aged black garlic water extract (ABGE) (1, 10, and 100 μg/mL) for 48 h, in basal (**a**) and after LPS pre-treatment (**b**) conditions. Data are reported as means ± SEM. ANOVA, Newman-Keuls multiple comparison post hoc test, * *p* < 0.05 vs. CTRL.

In the first series of experiments, we tested the effects of the ABGE (1–100 μg/mL) on the viability of cardiomyoblast (H9c2) cells. The experiments have been conducted both in basal conditions and after LPS-treatment for inducing an inflammatory status, in vitro. ABGE (1–100 µg/mL) did not alter H9c2 cell viability in basal conditions (Figure 2a). On the other hand, when H9c2 cells were treated with LPS, their viability was reduced, but ABGE (1–100 µg/mL) was able to revert the cytotoxicity (Figure 2b).

In particular, preclinical and clinical evidence has demonstrated that inflammation and oxidative stress play a crucial role in various CVD, including hypertension, fibrosis, diastolic dysfunction, left ventricular hypertrophy, heart failure, and ischemia/reperfusion damage [44].

Therefore, we investigated the protective effects induced by ABGE (1–100 μg/mL) in mouse heart specimens stimulated with LPS, which represents a validated model to study the modulatory activities of herbal extracts and drugs on inflammatory pathways and oxidative stress [33,34]. In particular, we evaluated the effects of ABGE (1–100 μg/mL) on pro-inflammatory and pro-oxidant mediators, such as COX-2, TNF-α, IL-6, NF-kB, and iNOS mRNA levels on isolated LPS-stimulated heart specimens, by RT-PCR analysis. This demonstrates the involvement of NF-κB in the transcription of various proinflammatory cytokines, such as TNF-α, and IL-6 [45], whose involvement in mediating cardiac dysfunction is well known [46].

**Figure 3:**
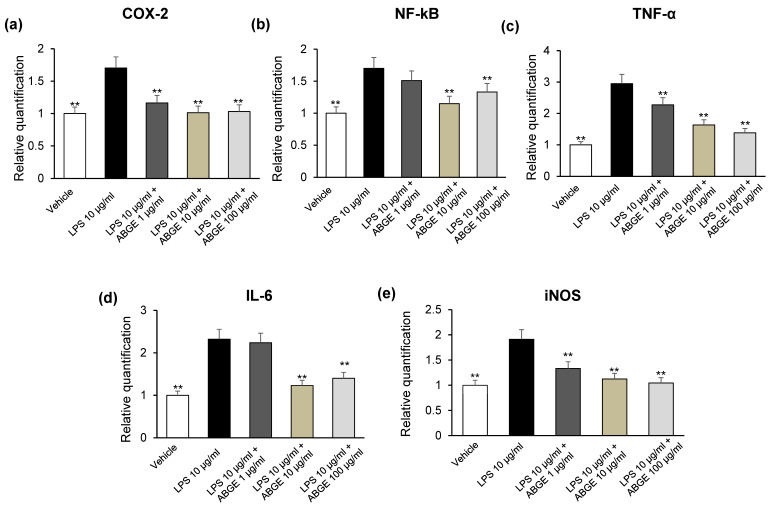
Effects of aged black garlic water extract (ABGE) (1, 10, and 100 μg/mL) on LPS-induced cyclooxygenase-2 (COX-2) (**a**), nuclear factor kB (NF-kB) (**b**), tumor necrosis factor α (TNF-α) (**c**), interleukin (IL)-6 (**d**), and inducible nitric oxide synthase (iNOS) (**e**) gene expression (RQ, relative quantification) in mouse heart specimens. Data are reported as means ± SEM. ANOVA, Newman-Keuls multiple comparison post hoc test. ** *p* < 0.005 vs. LPS.

In our ex vivo model, we observed that ABGE (10 and 100 μg/mL) significantly inhibited all markers investigated without showing a dose-dependent relationship (Figure 3a–e). In this context, polyphenol compounds have been suggested to induce cardioprotective effects by inhibiting oxidative stress and inflammation, as confirmed by a recently published study [7,47,48,49,50,51]. In particular, the beneficial activities induced by ABGE could be related to the pattern of polyphenolic composition, with particular regard to gallic acid and catechin. Accordingly, BenSaad and collaborators [52] (2017) reported that gallic acid inhibited LPS-induced prostaglandin E_2_ and IL-6 production in RAW264.7 cells. Gallic acid was hypothesized to be able to exert a protective effect on rat liver mitochondria by reducing oxidative stress induced by bisphenol A in ex vivo studies [53]. In addition, gallic acid pretreatment decreased levels of cardiac marker enzymes, including troponin T, which has been hypothesized to be involved in the myocardial damage reduction in rats [54]. Cardioprotective activities of catechins are also well known [55]. In particular, catechin administration attenuated coronary heart disease in a rat model by suppressing inflammation [56].

Moreover, catechin, as well as being known for its antioxidant activities, has been described as an anti-inflammatory agent, being able to inhibit COX-2 expression [57,58].

Furthermore, black garlic was found to exert stronger antioxidant activity than fresh garlic, as confirmed by in vivo and in vitro experiments [41].

**Figure 4:**
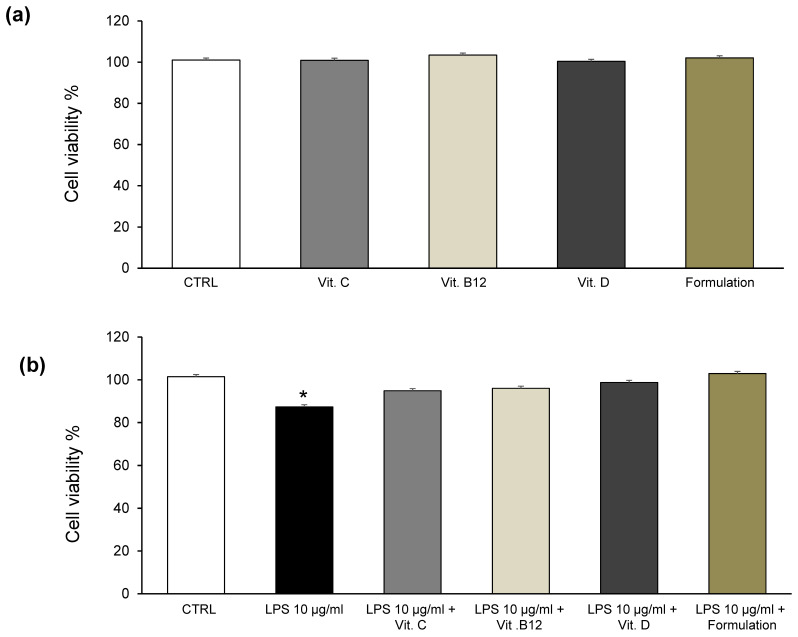
MTT assay of H9c2 cells exposed to Vitamin B12 (1 µg/mL), Vitamin C (10 µg/mL), vitamin D (1 µg/mL), and the Formulation [ABGE (100 µg/mL) + Vitamin B12 (1 µg/mL) + Vitamin C (10 µg/mL) + Vitamin D (1 µg/mL)] for 48 h in basal (**a**) and after LPS pre-treatment (**b**) conditions. Data are reported as means ± SEM. ANOVA, Newman-Keuls multiple comparison post hoc test * *p* < 0.05 vs. CTRL group.

On the basis of these results, we performed a second series of experiments, aimed at evaluating the effects of the Formulation [ABGE (100 µg/mL) + Vitamin B12 (1 µg/mL) + Vitamin C (10 µg/mL) + Vitamin D (1 µg/mL)] on the viability of LPS-pretreated and not LPS-pretreated H9c2 cells. The results were compared with vitamins alone [Vitamin B12 (1 µg/mL), Vitamin C (10 µg/mL), and vitamin D (1 µg/mL)]. Our findings showed that the Formulation and the vitamins alone did not modify H9c2 cell viability in basal conditions (Figure 4a). In addition, the Formulation and the vitamins alone were able to contrast the cytotoxicity induced by LPS in H9c2 cells (Figure 4b).

Thereafter, we investigated the effects induced by the Formulation and the vitamins alone on COX-2, TNF-α, NF-kB, IL-6, and iNOS mRNA levels in mouse heart specimens treated with LPS.

**Figure 5:**
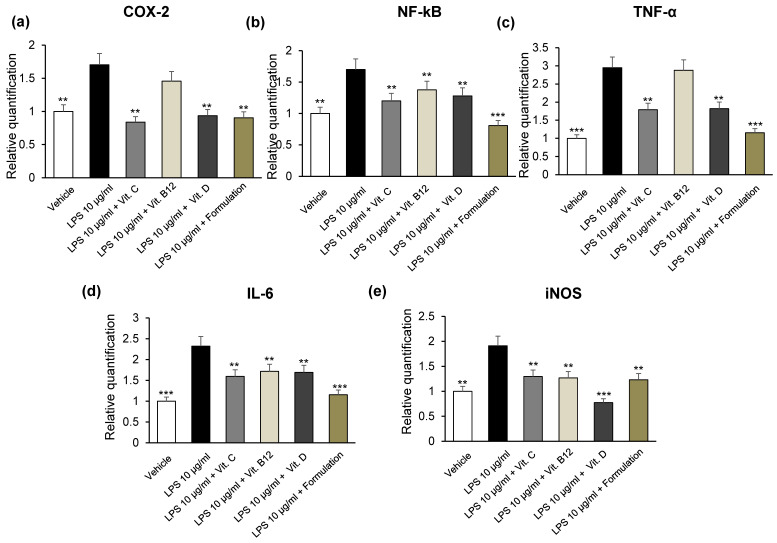
Effects of the Formulation [ABGE (100 µg/mL) + Vitamin B12 (1 µg/mL) + Vitamin C (10 µg/mL) + Vitamin D (1 µg/mL)] and the vitamins alone [Vitamin B12 (1 µg/mL), Vitamin C (10 µg/mL), Vitamin D (1 µg/mL)] on LPS-induced cyclooxygenase-2 (COX-2) (**a**), nuclear factor kB (NF-kB) (**b**), tumor necrosis factor-α (TNF-α) (**c**), interleukin (IL)-6 (**d**) and inducible nitric oxide synthase (iNOS) (**e**) gene expression (RQ, relative quantification) in mouse colon specimens. Data are reported as means ± SEM. ANOVA, Newman-Keuls multiple comparison post hoc test ** *p* < 0.005, *** *p* < 0.001 vs. LPS.

As shown in Figure 5a–e, vitamins C and D as well the Formulation reduced gene expression of almost all markers tested in our ex vivo study. In particular, the Formulation was more effective than vitamins alone in blunting LPS-induced gene expression of IL-6, TNF-α, and NF-kB. Recent studies reported that vitamin D represents one of the mediators playing a pivotal role in the pathogenesis of CVD [59]. In agreement, vitamin D supplementation was able to decrease inflammation and oxidative stress [59,60], confirming its pivotal role in heart tissue. In particular, TNF-α and IL-6 secretion was decreased by vitamin D in monocytes and macrophages [61]. Vitamin D also exerts various potent antioxidant effects by downregulating intracellular oxidative stress-related protein oxidation, lipid peroxidation, and DNA damage [62].

Similarly, an inverse correlation between vitamin C supplementation and the risk of CVD has been suggested in various observational studies [63,64]. In this regard, the antioxidant effects of vitamin C have been shown to be involved in both prevention and treatment of CVD [65]. In particular, Ellulu [62] (2017) showed that vitamin C protected against oxidative stress via its effect on nitric oxide release as well as alleviating inflammation by down-regulating IL-6, TNF-α, and NF-kB mRNA levels [66]. As for vitamin B12, its deficiency can cause hyperhomocysteinemia, an independent risk factor for CVD [27]. Moreover, an association between vitamin B12 deficiency and increased incidence of inflammation and associated metabolic complications has been demonstrated by a number of studies [67,68]. Our present findings showed that vitamin B12 decreased LPS-induced gene expression of NF-kB, IL-6, and iNOS. In agreement, Birch and collaborators [69] (2009) showed that vitamin B12 decreased NF-kB levels, which could represent a signaling molecule of vitamin B12 deficiency. Moreover, vitamin B12 was able to suppress IL-6 production, in vitro. Weinberg et al. [70] (2009) also reported that vitamin B12 is involved in the modulation of NOS function and NO synthesis in vivo.

### 3.4. Evaluation of H_2_S Release in HASMCs

**Figure 6:**
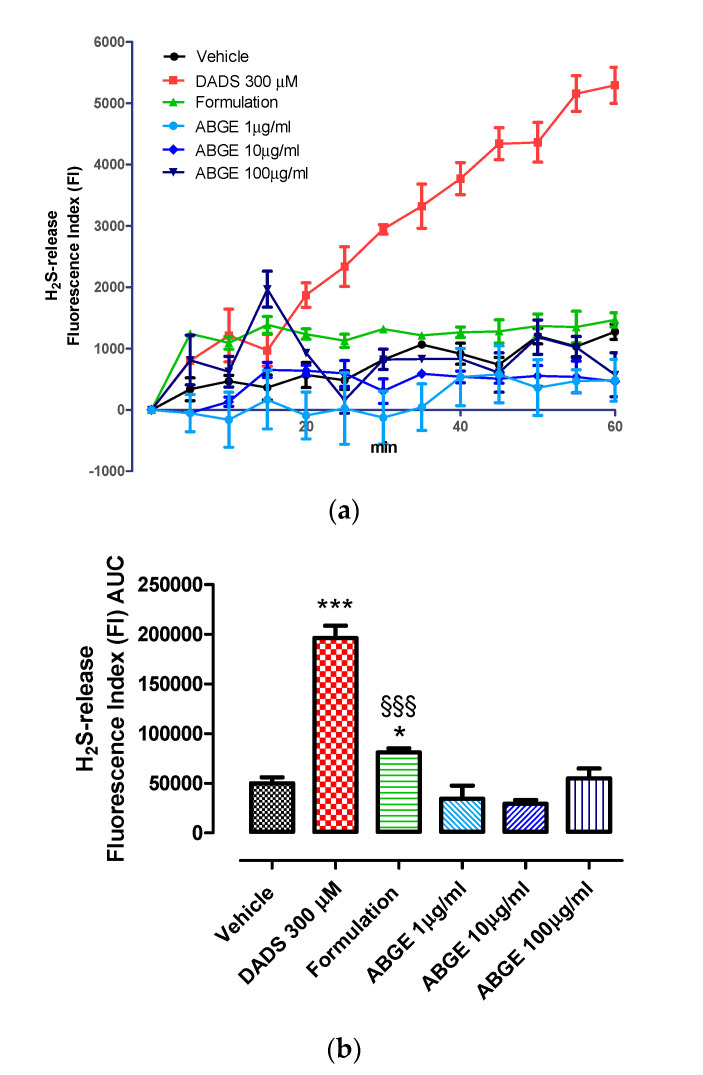
Fluorometric recording of H_2_S-release inside HASMCs. (**a**) Time course of the fluorometric recordings of H_2_S released by vehicle, ABGE (1–100 μg/mL), Formulation [ABGE (100 µg/mL) + Vitamin B12 (1 µg/mL) + Vitamin C (10 µg/mL) + Vitamin D (1 µg/mL)] and DADS 300 μM, during 60 min of observation: the increase in H_2_S is expressed as FI. (**b**) The histograms show the total amount of H_2_S released by vehicle, ABGE (1–100 μg/mL), Formulation [ABGE (100 µg/mL) + Vitamin B12 (1 µg/mL) + Vitamin C (10 µg/mL) + Vitamin D (1 µg/mL)] and DADS 300 μM in the 60 min of observation time, expressed as AUC. The vertical bars represent SEM, six different experiments were performed, each with six replicates (n = 6). ANOVA, Bonferroni post hoc test * *p* < 0.05, *** *p* < 0.001 vs. vehicle; §§§ *p* < 0.001 vs. ABGE.

H_2_S has been suggested to be able to modulate many pathways related to cardiovascular pathophysiology [71]. In addition, it is one of the most important biological mediators involved in different pathological processes, where inflammation plays a predominant role, including CVD [72]. H_2_S is known to be critically involved in garlic-induced cardioprotective effects [73,74,75]. In this context, H_2_S was shown to play a key role in preventing the progression of cardiac hypertrophy to heart failure [76]. Considering the inhibitory effects induced by both ABGE (1–100 µg/mL) and the Formulation [ABGE (100 µg/mL) supplemented with Vitamin B12 (1 µg/mL) + Vitamin C (10 µg/mL) + Vitamin D (1 µg/mL)] on the investigated markers of inflammation and oxidative stress in our study, we also evaluated their potential effects on H_2_S releasing properties using cultured HASMCs. DADS (300 µM) was used as a known H_2_S releasing molecule and significantly increased the fluorescence index, thus indicating the intracellular H_2_S formation (Figure 6a,b). We showed that ABGE did not determine significant H_2_S formation into the cells with respect to the vehicle (Figure 6a,b). Our findings are in agreement with those of Leitao et al. (2022) [77], showing that improvement of microvascular reactivity induced by aged garlic extract was not mediated by H_2_S in older adults at CVD risk. Interestingly, the Formulation significantly increased fluorescence values compared to the vehicle, reflecting the H_2_S formation inside the cells. In this context, we speculate that the presence of the vitamins into the Formulation allows the garlic extract to more easily cross the cell membrane and release H_2_S. In this regard, B vitamins could act as cofactors of enzymes playing a key role in the sulfur network and modulate H_2_S production [78]. Accordingly, Wilinski et al. [79] (2012) showed that vitamin D increased H_2_S levels in a number of mouse organs, including the heart [79].

An important finding of our study is that the Formulation tested has increased the release of H_2_S, suggesting its potential role on CVD, including hypertension, thanks to its vasodilatation action [80]. In addition to its vasoprotective effects, H_2_S could be critically involved in the pathogenesis of hypertension-related vascular dysfunction through its effects on blood pressure regulation, too, as well as inflammation [81,82,83,84].

### 3.5. Evaluation of Membrane Hyperpolarization of HASMCs

**Figure 7:**
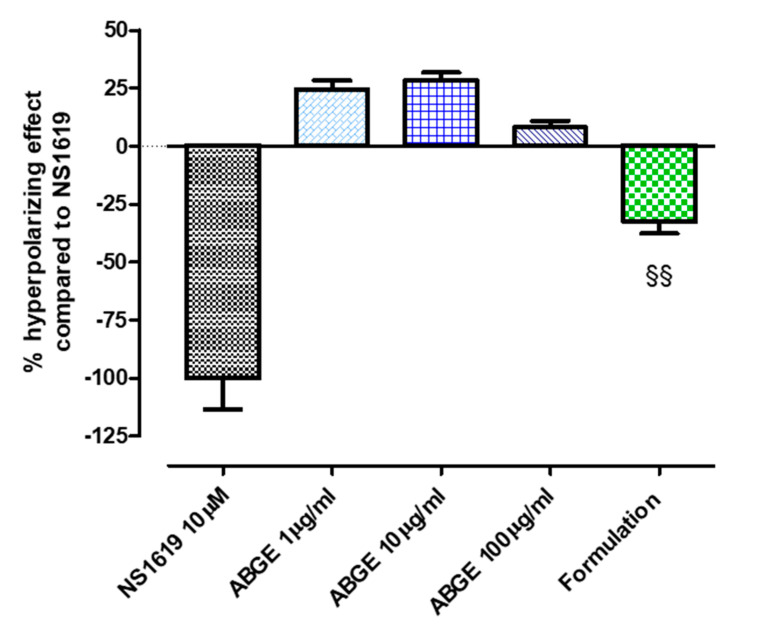
Hyperpolarizing effects in HASMCs. % hyperpolarization calculated as mean of changes in HASMCs membrane potential, followed for 35 min, induced by ABGE (1–100 µg/mL) or the Formulation [ABGE (100 µg/mL) + Vitamin B12 (1 µg/mL) + Vitamin C (10 µg/mL) + Vitamin D (1 µg/mL)]. Data are expressed as mean ± SEM, reported as % of the hyperpolarizing effect evoked by (1,3-dihydro-1-[2-hydroxy-5-(trifluoromethyl)phenyl]-5-(trifluoromethyl)-2H-benzimidazole-2-one (NS1619). Six different experiments were performed, each with six replicates (*n* = 6). The §§ indicates a significant difference from the effect evoked by ABGE (ANOVA, Bonferroni post hoc test §§ *p* < 0.01).

In addition, the effects of ABGE and the Formulation were evaluated on the membrane potential of cultured HASMCs. We showed that ABGE (1–100 μg/mL) did not modify membrane hyperpolarization. On the other hand, we showed that the Formulation caused a significant membrane hyperpolarization of HASMCs compared to ABGE (1–100 μg/mL) (Figure 7).

These results seem to suggest that the hyperpolarization is a consequence of the ability of the Formulation to release H_2_S. Indeed, it is well known that compounds able to release H_2_S, also called as H_2_S-donors, exhibited the property to induce vascular smooth muscle hyperpolarization through the activation of different subtypes of potassium channels [36,85].

Experimental and clinical studies showed that ABG was able to exert beneficial effects on cardiometabolic alterations, which are usually related to metabolic syndrome [86,87]. Accordingly, Amor and collaborators [22] (2019) showed an improvement of metabolic syndrome following ABG treatment in rats [22]. In this context, the aging process was suggested to enhance the activity of bioactive compounds, including S-allylcysteine and S-allylmercaptocysteine, whose cardioprotective effects are well known [88,89]. It is also well known that black garlic shows a reduced content of allicin when subjected to high temperatures during the production phase [87]. Moreover, Bradley and collaborators [73] (2016) suggested that allicin and alliin could not be the main bioactive compounds involved in the cardioprotective effects induced by aged garlic.

The content of phytochemicals in garlic has also been reported to be dependent on environmental, genetic, and agronomic factors [90].

Interestingly, gallic acid was found able to induce hyperpolarization of the cell membranes and excitation of muscles by binding to glutamate-gated chloride channels [91]. Furthermore, catechins have also been suggested to display inhibitory effects on voltage-dependent Ca^2+^ channels involving, albeit partially, membrane hyperpolarization deriving from the opening of  K^+^ channels [92]. Finally, we hypothesized that the beneficial effects of the Formulation are due to the presence of gallic acid, catechin, and vitamins.

In conclusion, our results showed that ABGE, alone and in association with multivitamins consisting of combined Vitamins D, C, and B12, exhibited protective effects, as confirmed by the inhibitory activities on multiple inflammatory and oxidative stress-related pathways on mouse heart specimens exposed to LPS. These effects could be related, at least in part, to the ABGE content in polyphenolic compounds, with particular regards to gallic acid and catechin. Moreover, the Formulation increased intracellular H_2_S formation, and caused a significant membrane hyperpolarization of HASMCs, further suggesting its potential use on CVD. In this context, we speculate that the presence of the vitamins in the Formulation allows the garlic extract to more easily cross the cell membrane and release H_2_S. However, further studies using independent experimental paradigms are necessary to accurately evaluate the in vivo activity.

## Figures and Tables

**Table 1:** Retention times, *m/z* ratio, as well as quantity (g/mL) of the investigated phenolic compounds in ABGE.

Peak Name	tR	m/z (Positive Ion)	Quantity (μg/mL)
Gallic Acid	8.80	171.12	24.495
3-Hydroxytirosol	11.71	155.16	1.713
Caftaric acid	12.93	313.23	1.773
Catechin	14.80	291.26	30.877
Gentisic acid	15.82	155.12	1.322
4-Hydroxybenzoic acid	16.20	193.12	n/a
Loganic acid	16.60	377.36	n/a
Chlorogenic acid	16.81	355.31	0.561
Vanillic acid	18.60	169.14	n/a
Caffeic acid	19.00	181.16	1.153
Epicatechin	19.41	291.26	1.338
Syringic acid	21.80	183.17	1.724
p-Coumaric acid	23.06	165.16	n/a
t-Ferulic acid	24.00	195.18	n/a
Benzoic acid	26.38	123.12	3.891
Rutin	27.16	611.52	n/a
Resveratrol	27.70	229.25	1.058

## Data Availability

The datasets used and/or analyzed during the current study are available from the corresponding author on reasonable request.

## Supplementary Materials

The following supporting information can be downloaded at: https://www.mdpi.com/article/10.3390/foods12071558/s1, Table S1. Gradient Elution Conditions; Table S2. MS analysis conditions.
