# Supplementary material for: Anti-Inflammatory and Vasorelaxant Effects Induced by an Aqueous Aged Black Garlic Extract Supplemented with Vitamins D, C, and B12 on Cardiovascular System
Source: Foods. 2023 Apr 6;12(7):1558. doi: 10.3390/foods12071558 (PMC10094181; doi:10.3390/foods12071558)
Supplement: Supplementary file 1 [file foods-12-01558-s001.zip › foods-2266577-supplementary.pdf]

Type of the Paper (Article, Review, Communication, etc.)

Anti-inflammatory and vasorelaxant effects induced by an aqueous aged black garlic extract supplemented with vitamins D, C and B12 on cardiovascular system.

Lucia Recinella [1,†], Maria Loreta Libero [1,†,], Valentina Citi [2], Annalisa Chiavaroli [1,*], Alma Martelli [2,3,4,*], Roberta Foligni [5], Cinzia Mannozzi [5], Alessandra Acquaviva [1,6], Simonetta Di Simone [1], Vincenzo Calderone [2,3,4], Giustino Orlando [1], Claudio Ferrante [1], Serena Veschi [1], Anna Piro [1], Luigi Menghini [1], Luigi Brunetti [1] and Sheila Leone [1]

[1] Department of Pharmacy, G. d'Annunzio University of Chieti-Pescara, 66013, Chieti, Italy; lucia.recinella@unich.it (L.R.); maria.libero@unich.it (M.L.); annalisa.chiavaroli@unich.it (A.C.); alessandra.acquaviva@unich.it (A.A.); simonetta.disimone@unich.it (S.D.S.); giustino.orlando@unich.it (G.O.); claudio.ferrante@unich.it (C.F.); veschi@unich.it (S.V.); anna.piro@unich.it (A.P.); luigi.brunetti@unich.it (L.B.); sheila.leone@unich.it (S.L.).

[2] Department of Pharmacy, University of Pisa, 56126, Pisa, Italy; Valentina Citi valentina.citi@unipi.it (V.C.); alma.martelli@unipi.it (A.M.); vincenzo.calderone@unipi.it (V.C.).

[3] Interdepartmental Research Center "Nutrafood: Nutraceutica e Alimentazione per la Salute", University of Pisa, Italy; alma.martelli@unipi.it (A.M.); vincenzo.calderone@unipi.it (V.C.).

[4] CISUP, Centre for Instrumentation Sharing of Pisa University, 56126, Pisa, Italy; Valentina Citi email valentina.citi@unipi.it (V.C.); alma.martelli@unipi.it (A.M.); vincenzo.calderone@unipi.it (V.C.).

[5] Department of Agricultural, Food and Environmental Sciences, Polytechnic University of Marche, Via Brecce Bianche 10, 60121 Ancona, Italy; r.foligni@staff.univpm.it (R.F.); c.mannozzi@staff.univpm.it (C.M.)

[6] Veridia Italia Srl, Via Piano di Sacco – 65013, Città Sant'Angelo (PE), Italy; alessandra.acquaviva@unich.it

* Correspondence: maria.libero@unich.it; Tel.: +39-0871-3554673; sheila.leone@unich.it; Tel.: +39-0871-3554658.

[#] These authors equally contributed to the manuscript.

Citation: Recinella, L.; Libero, M.L.; Citi, V.; Chiavaroli, A.; Martelli, A.; Foligni, R.; Mannozzi, C.; Acquaviva, A.; Di Simone, S.; Calderone, V.; et al. Anti-Inflammatory and Vasorelaxant Effects Induced by an Aqueous Aged Black Garlic Extract Supplemented with Vitamins D, C and B12 on Cardiovascular System. *Foods* **2023**, *12*, x. https://doi.org/10.3390/xxxxx

Academic Editor(s): Sonia Medina

Received: 21 February 2023
Revised: 27 March 2023
Accepted: 30 March 2023
Published: date

Copyright: © 2023 by the authors. Submitted for possible open access publication under the terms and conditions of the Creative Commons Attribution (CC BY) license (https://creativecommons.org/licenses/by/4.0/).

2. Materials and Methods

2.1 Preparation of ABGE

ABG cloves were supplied as dried material by il Grappolo S.r.l. (Soliera, Modena, Italy). 10 g of garlic cloves were crushed with a utility garlic crusher by hand, and the juice and debris of the garlic were collected in a centrifuge tube by pouring 10 mL of water onto the crusher.

After having been shaken 10 times, the tube was placed in a Trans-sonic T460 ultrasonic bath supplied by Elma (Singen, Germany) for 15 min at room temperature and then centrifuged twice at 4000 rpm for 10 min each time [1-2]. The supernatant was filtered, until chemical analyses were performed.

2.2 Total polyphenol content of ABGE

Total polyphenol content was determined according to the Folin-Ciocalteu method, as described in Savini et al. [3] (2017) with some modifications. Briefly, in a 50 ml volumetric flask, 1 ml of extract was added to 0.5 ml of Folin-Ciocalteu reagent 2 N (Sigma-Aldrich, St. Louis, MO), 2 ml of sodium carbonate (Sigma-Aldrich, St. Louis, MO) 15% w/v solution, and made up to volume with water. The reaction mixture was allowed to stand for 2 hours before the absorbance at 765 nm was measured against a blank of reagents and solvents. A set of working

solutions (50, 100, 300, 500, and 700 ppm) of gallic acid (Sigma-Aldrich, Milan, Italy) was used for the calibration curve. The total amount of the phenolic compounds was expressed as milligrams of gallic acid equivalents (GAE) per gram of dry weight.

2.3 HPLC-DAD-MS analysis of phenolic compounds

The extract was analyzed for phenol quantitative determination using a reversed-phase HPLC-DAD-MS in gradient elution mode [4]. The separation was conducted within 60 min of the chromatographic run, starting from the following separation conditions: 97 % water with 0.1 % formic acid, 3 % methanol with 0.1 % formic acid. The separation was performed on an Infinity lab Poroshell 120-SB reverse phase column (C18, 150 × 4.6 mm i.d., 2.7 μm; Agilent, Santa Clara, CA, USA). Column temperature was set at 30 °C. Quantitative determination of phenolic compounds was performed via a DAD detector. Quantification was done through 7-point calibration curves, with linearity coefficients (R2) > 0.999, in the concentration range 2–140 μg/mL. All standards were purchased from Sigma Aldrich (Milan, Italy), and have a purity ≥ 95 %. The limits of detection were lower than 1 μg/mL for all assayed analytes. The area under the curve from HPLC chromatograms was used to quantify the analyte concentrations in the extract. The extracts were also qualitatively analyzed using an expression compact mass spectrometer (Advion, Ithaca, NY, USA) in negative and positive ion mode (m/z scan mode: 100–1200) [4]. MS signal identification was realized through comparison with a standard solution and MS spectra present in the MassBankEurope database. The details of the analysis are reported in supplementary materials (Tables S1-S2).

Table S1. Gradient Elution Conditions

TIME (Min.)	COMPOSITION A% (Water+Formic acid 0.1%)	COMPOSITION B% (Methanol+Formic acid 0.1%)	FLOW (mL/min)
**1.00**	97.0	3.0	0.600
**5.00**	77.0	23.0	0.600
**12.00**	73.0	27.0	0.600
**18.00**	57.0	43.0	0.600
**25.00**	52.0	48.0	0.600
**32.00**	50.0	50.0	0.600
**34.00**	50.0	50.0	0.600
**37.00**	35.0	65.0	0.600
**40.00**	5.0	95.0	0.600
**47.00**	5.0	95.0	0.600
**48.00**	97.0	3.0	0.600
**60.00**	97.0	3.0	0.600

Table S2. MS analysis conditions

Ion Source Parameters (APCI Positive):

Capillary Temperature = 250 °C

Capillary Voltage = 140 V
Source Voltage Offset = 30 V
Source Voltage Span = 10 V
APCI Source:
Source Gas Temperature = 250 °C
Transfer Line Temperature = 100 °C
APCI Corona Discharge = 3 μA
Tune Parameters Voltages:
Extraction Electrode = 9
Hexapole Bias = 8
Hexapole RF Offset = 5
Hexapole RF Span = 150
Ion Energy Offset = -1.2
Ion Energy Span = -0.4
Resolution Offset= -0.02
Resolution Span = -1.7
Detector gain = 1200

2.4 *Toxicological and pharmacological studies*

2.4.1. Cell line

H9c2 cells (rat cardiomyoblasts, ATTC, USA) were maintained in DMEM (Sigma-Aldrich, USA) supplemented with 10% fetal bovine serum (FBS), 1% of 100 unit/ml penicillin and 100 mg/mL streptomycin (Sigma-Aldrich, USA) in T75 red cap tissue culture flasks, at 37 °C in a humidified atmosphere of 5% CO_2.

2.4.2. Cell viability assay

Cell viability was evaluated by MTT assay [3-(4,5-dimethyl-2-thiazolyl)-2,5-diphenyl-2H-tetrazolium bromide] (Sigma, St. Louis, MO, USA) as previously described [5]. Briefly, H9c2 cell line was seeded in 96-well plates ($5x10^3$ cells/well) and it was pretreated with 10 μg/ml LPS for 24 hours. Subsequently, both LPS-pretreated and not LPS-pretreated H9c2 cells were exposed to ABGE at various concentrations (1-100 μg/ml), or with vehicle (control) for a further 48 hours. Then, on the basis of results, we performed a second set of experiments to evaluate the effects induced by the Formulation [ABGE (100 μg/ml) + Vitamin B12 (1 μg/ml) + Vitamin C (10 μg/ml) + Vitamin D (1 μg/ml)] and the vitamins alone [Vitamin B12 (1 μg/ml), Vitamin C (10 μg/ml) and vitamin D (1μg/ml)] on H9c2 cell viability in both LPS- and not LPS-pretreatment.

After treatment, the MTT solution was added to each well and incubated at 37 °C for at least 3 hours, until purple formazan crystals were formed. In order to dissolve the precipitate, the culture medium was replaced with dimethyl sulfoxide (DMSO, Euroclone). Absorbance of each well was quantified at 540 and 690 nm, using a Synergy H1 microplate reader (BioTek Instruments Inc., Winooski, VT, USA) [5].

2.4.3. *Ex vivo* studies

Adult C57/BL6 male mice (3-month-old, weight 20–25 g) were housed in Plexiglas cages (2–4 animals per cage; 55 cm x 33 cm x 19 cm) and maintained under standard laboratory conditions (21 ± 2 °C; 55 ± 5% humidity) on a 14/10 h light/dark cycle, with ad libitum access to water and food. Housing conditions and experimentation procedures were strictly in agreement with the European Community ethical regulations (EU Directive no. 26/2014) on the care of animals for scientific research. In agreement with the recognized principles of "Replacement, Refinement and Reduction in Animals in Research", heart specimens were obtained as residual material from vehicle-treated mice randomized in our previous experiments, approved by local ethical committee

('G. d'Annunzio' University, Chieti, Italy) and Italian Health Ministry (Project no. 885/2018-PR).

After collection, isolated heart specimens were maintained in a humidified incubator with 5% CO_2 at 37 °C for 4 h (incubation period), in RPMI buffer with added bacterial LPS (10 μg/mL), as previously described [6-7]. During the incubation period, the tissues were challenged with scalar concentrations of ABGE (1μg/ml, 10 μg/ml, 100 μg/ml), the Formulation [ABGE (10 μg/ml) + Vitamin B12 (1 μg/ml) + Vitamin C (10 μg/ml) + Vitamin D (1 μg/ml)] and the vitamins alone [Vitamin B12 (1 μg/ml), Vitamin C (10 μg/ml) and vitamin D (1μg/ml)].

Total RNA was extracted from the heart specimens using TRI Reagent (Sigma–Aldrich, St. Louis, MO, USA), according to the manufacturer's protocol. Contaminating DNA was removed using 2 units of RNase-free DNase 1 (DNA-free kit, Ambion, Austin, TX, USA). The RNA concentration was quantified at 260 nm by spectrophotometer reading (BioPhotometer, Eppendorf, Hamburg, Germany) and its purity was assessed by the ratio at 260 and 280 nm readings. The quality of the extracted RNA samples was also determined by electrophoresis through agarose gels and staining with ethidium bromide, under UV light. One microgram of total RNA extracted from each sample in a 20 μL reaction volume was reverse transcribed using High Capacity cDNA Reverse Transcription Kit (Thermo Fisher Scientific Inc., Monza, Italy). Reactions were incubated in a 2720 Thermal Cycler (Thermo Fisher Scientific Inc., Monza, Italy) initially at 25 °C for 10 min, then at 37 °C for 120 min, and finally at 85 °C for 5 s. Gene expression of COX-2, IL-6, NF-kB, TNF-α and iNOS was determined by quantitative real-time PCR using TaqMan probe-based chemistry, as previously described [8]. PCR primers and TaqMan probes, including β-actin used as the housekeeping gene, were purchased from Thermo Fisher Scientific Inc. (Assays-on-Demand Gene Expression Products, Mm00478374_m1 for COX-2 gene, Mm00443258_m1 for TNF-α gene, Mm00446190_m1 for IL-6 gene, Mm00476361_m1 for NF-kB gene, Mm00440502_m1 for iNOS gene, Mm00607939_s1 for β-actin gene). The real-time PCR was carried out in triplicate for each cDNA sample in relation to each of the investigated genes. Data were elaborated with the Sequence Detection System (SDS) software version 2.3 (Thermo Fisher Scientific Inc.). Gene expression was relatively quantified by the comparative $2^{-\Delta\Delta Ct}$ method method [9].

2.4.4. Cell line

HASMCs were cultured in Medium 231 (Life technologies) supplemented with Smooth Muscle Growth Supplement (SMGS, Life Technologies) and 1% of 100 units/ml penicillin and 100 mg/ml streptomycin (Sigma Aldrich) in tissue culture flasks at 37 °C in a humidified atmosphere and 5% CO_2. HASMCs were cultured up to about 90% confluence and 24 h before the experiment cells were seeded onto a 96-well black plate, clear bottom pre-coated with gelatin 1% (from porcine skin, Sigma Aldrich), at density of 72 × 10^3 per well for the evaluation of the membrane hyperpolarizing effects, at density of 30 × 10^3 per well for the evaluation of H_2S release. Cells were split 1:2 twice a week and used until passage 18.

2.4.5. Evaluation of H_2S release on HASMCs

After 24 h to allow cell attachment, the medium was replaced and cells were incubated for 30 min in the buffer standard (HEPES 20 mM, NaCl 120 mM, KCl 2 mM, $CaCl_2{\cdot}2H_2O$ 2 mM, $MgCl_2{\cdot}6H_2O$ 1 mM, Glucose 5 mM, pH 7.4, at room temperature) containing the fluorescent dye WSP-1 (Washington State Probe-1, 3′-methoxy-3-oxo-3H-spiro(isobenzofuran-1,9′-xanthen)-6′yl2(pyridin-2-yldisulfanyl) benzoate, Cayman Chemical) at the concentration of 100 μM [10].

Then the supernatant was removed and replaced with a solution of the ABGE (1μg/ml, 10 μg/ml, 100 μg/ml), or the Formulation 10 μg/ml, or DADS 300 μM (used as reference drugs as well-known H_2S-donor), all dissolved in buffer standard containing dimethylsulfoxide (DMSO) 0,1% (vehicle). When WSP-1 reacts with H_2S, it releases a fluorophore detectable with a spectrofluorometer at excitation and emission wavelengths of 465-515 nm [11]. The increasing of fluorescence (expressed as fluorescence index=FI) was monitored for 60 min, using a spectrofluorometer (EnSpire, Perkin Elmer). The obtained curves were analyzed to obtain the area under the curve (AUC).

2.4.6. Evaluation of the membrane hyperpolarizing effects on HASMCs

After 24 h to allow cell attachment, the medium was replaced and cells were incubated for 1 h in the buffer standard (HEPES 20 mM, NaCl 120 mM, KCl 2 mM, $CaCl_2{\cdot}2H_2O$ 2 mM, $MgCl_2{\cdot}6H_2O$ 1 mM, Glucose 5 mM, pH 7.4, at room temperature) containing the bisoxonol dye bis-(1,3-dibutylbarbituric acid) DiBac4(3) (Sigma Aldrich) 2.5 μM [12]. This membrane potential-sensitive dye DiBac4(3) measures the cell membrane potential, shuffling between cellular and extracellular fluids in a membrane potential-dependent manner (following the Nernst laws). In particular, an increase of fluorescence, corresponding to an inward flow of the dye, reflects a membrane depolarization; in contrast, a decrease in fluorescence, due to an outward flow of the dye, is linked to membrane hyperpolarization. The spectrofluorometric recording is carried out at excitation and emission wavelengths of 488 and 520 nm, respectively (Multiwells reader, Enspire, PerkinElmer). NS1619 (Sigma-Aldrich) 10 μM, a BK_{Ca} channel opener, was used as reference drug. The ABGE (1-100 μg/ml), or the Formulation and the vitamins alone (Vitamin B12 1 μg/ml, Vitamin C 10 μg/ml and Vitamin D 1μg/ml) were added to the cells, and the trends of fluorescence was followed for 35 min. The relative fluorescence decrease, linked to hyperpolarizing effects, was recorded every 2.5 min and was calculated as $(F_t{-}F_0)/F_0$ where F_0 is the basal fluorescence before the addition of the tested compounds, and F_t is the fluorescence at time t after their administration. Moreover, the mean of the hyperpolarizing effect was calculated. Data are expressed as a % of the hyperpolarizing effects induced by NS1619 10 μM. Six different experiments (n = 6) were performed.

2.5 Statistical analysis

The software GraphPad Prism version 6.0 (Graphpad Software Inc., San Diego, CA, USA) was used to perform analysis of the data. Means ± SEM were determined for each experimental group and analyzed by one-way analysis of variance (ANOVA), followed by Newman-Keuls multiple comparison post hoc test and by Bonferroni post hoc test. The limit of statistically significant differences between mean values was set at p-value < 0.05. The number of animals randomized for each experimental group was calculated on the basis of the "Resource Equation" $N = (E + T)/T$ ($10 \le E \le 20$) [13]. The Tukey-Kramer's Honest Significant Difference (HSD) test was used to compare the mean polyphenol contents of the extracts. The level of significance was set to 0.05.

References

1. Fujisawa, H.; Suma, K.; Origuchi, K.; Kumagai, H.; Seki, T.; Ariga, T. Biological and Chemical Stability of Garlic-Derived Allicin. *J. Agric. Food Chem.* **2008**, *56*, 4229–4235, doi:10.1021/jf8000907.
2. Fujisawa, H.; Suma, K.; Origuchi, K.; Seki, T.; Ariga, T. Thermostability of Allicin Determined by Chemical and Biological Assays. *Biosci., Biotech., Biochem.* **2008**, *72*, 2877–2883, doi:10.1271/bbb.80381.
3. Savini, S.; Loizzo, M.R.; Tundis, R.; Mozzon, M.; Foligni, R.; Longo, E.; Morozova, K.; Scampicchio, M.; Martin-Vertedor, D.; Boselli, E. Fresh Refrigerated Tuber Melanosporum Truffle: Effect of the Storage Conditions on the

Antioxidant Profile, Antioxidant Activity and Volatile Profile. *Eur. Food Res. Technol.* **2017**, *243*, 2255–2263, doi:10.1007/s00217-017-2927-x.
4. Acquaviva, A.; Cristina Di Simone, S.; Canini, A.; Braglia, R.; Di Marco, G.; Campana, C.; Angelini, P.; Angeles Flores, G.; Venanzoni, R.; Loreta Libero, M.; Tirillini, B.; Zengin, G.; Chiavaroli, A.; Recinella, L.; Leone, S.; Nilofar, Brunetti, L.; Orlando, G.; Menghini, L.; Ferrante, C. Phytochemical and Biological Investigations on the Pollen from Industrial Hemp Male Inflorescences. *Food Res. Int.* **2022**, *161*, 111883, doi:10.1016/j.foodres.2022.111883.
5. Florio, M.; Heide, M.; Pinson, A.; Brandl, H.; Albert, M.; Winkler, S.; Wimberger, P.; Huttner, W.B.; Hiller, M. Evolution and Cell-Type Specificity of Human-Specific Genes Preferentially Expressed in Progenitors of Fetal Neocortex. *eLife* **2018**, *7*, e32332, doi:10.7554/eLife.32332.
6. Recinella, L.; Chiavaroli, A.; Masciulli, F.; Fraschetti, C.; Filippi, A.; Cesa, S.; Cairone, F.; Gorica, E.; De Leo, M.; Braca, A.; Martelli, A; Calderone, V; Orlando, G; Ferrante, C; Menghini, L; Di Simone, S.C.; Veschi, S.; Cama, A.; Brunetti, L.; Leone, S. Protective Effects Induced by a Hydroalcoholic Allium Sativum Extract in Isolated Mouse Heart. *Nutrients* **2021**, *13*, 2332, doi:10.3390/nu13072332.
7. Recinella, L.; Chiavaroli, A.; Orlando, G.; Ferrante, C.; Diletta Marconi, G.; Gesmundo, I.; Granata, R.; Cai, R.; Sha, W.; Schally, A.V.; Brunetti, L.; Leone, S. Antinflammatory, Antioxidant, and Behavioral Effects Induced by Administration of Growth Hormone-Releasing Hormone Analogs in Mice. *Sci. Rep.* **2020**, *10*, 732, doi:10.1038/s41598-019-57292-z.
8. Recinella, L.; Chiavaroli, A.; Orlando, G.; Menghini, L.; Ferrante, C.; Di Cesare Mannelli, L.; Ghelardini, C.; Brunetti, L.; Leone, S. Protective Effects Induced by Two Polyphenolic Liquid Complexes from Olive (Olea Europaea, Mainly Cultivar Coratina) Pressing Juice in Rat Isolated Tissues Challenged with LPS. *Molecules* **2019**, *24*, 3002, doi:10.3390/molecules24163002.
9. Livak, K.J.; Schmittgen, T.D. Analysis of Relative Gene Expression Data Using Real-Time Quantitative PCR and the 2−ΔΔCT Method. *Methods* **2001**, *25*, 402–408, doi:10.1006/meth.2001.1262.
10. Martelli, A.; Piragine, E.; Citi, V.; Testai, L.; Pagnotta, E.; Ugolini, L.; Lazzeri, L.; Di Cesare Mannelli, L.; Manzo, O.L.; Bucci, M.; Ghelardini, C.; Breschi, M.C.; Calderone, V. Erucin Exhibits Vasorelaxing Effects and Antihypertensive Activity by H2S-Releasing Properties. *British J. Phar.* **2020**, *177*, 824–835, doi:10.1111/bph.14645.
11. Peng, B.; Chen, W.; Liu, C.; Rosser, E.W.; Pacheco, A.; Zhao, Y.; Aguilar, H.C.; Xian, M. Fluorescent Probes Based on Nucleophilic Substitution–Cyclization for Hydrogen Sulfide Detection and Bioimaging. *Eur. J. Chem.* **2014**, *20*, 1010–1016, doi:10.1002/chem.201303757.
12. Martelli, A.; Citi, V.; Calderone, V. Vascular Effects of H2S-Donors: Fluorimetric Detection of H2S Generation and Ion Channel Activation in Human Aortic Smooth Muscle Cells. *Methods Mol. Biol.* **2019**, *2007*, 79–87, doi: 10.1007/978-1-4939-9528-8_6
13. Charan, J.; Kantharia, N.D. How to Calculate Sample Size in Animal Studies? *J. Pharmacol. Pharmacother.* **2013**, *4*, 303–306, doi:10.4103/0976-500X.119726.
